# Improvement of the Interaction Model Aimed to Reduce the Negative Effects of Cybersickness in VR Rehab Applications

**DOI:** 10.3390/s21020321

**Published:** 2021-01-06

**Authors:** Predrag Veličković, Miloš Milovanović

**Affiliations:** Faculty of Organisational Sciences, Jove Ilića 154, 11000 Belgrade, Serbia; milos.milovanovic@mmklab.org

**Keywords:** virtual reality, cybersickness, HCI, rehabilitation

## Abstract

Virtual reality (VR) has the potential to be applied in many fields, including medicine, education, scientific research. The e-health impact of VR on medical therapy for people cannot be ignored, but participants reported problems using them, as the capabilities and limitations of users can greatly affect the effectiveness and usability of the VR in rehabilitation. Previous studies of VR have focused on the development and use of the technology itself, and it is only in recent years that emphasis has been placed on usability problems that include the human factor. In this research, different ways of adapting interaction in VR were tested. One approach was focused on means of navigating through a VR, while the second dealt with the impact of the amount of animation and moving elements through a series of tests. In conclusion, the way of navigation and the amount of animation and moving elements, as well as their combination, are proven to have a great influence on the use of VR systems for rehabilitation. There is a possibility to reduce the occurrence of problems related to cybersickness if the results of this research are taken into consideration and applied from an early stage of designing VR rehabilitation applications.

## 1. Introduction

Virtual reality (VR) technology has advanced in recent years and there is an increasing number of areas in which it can be used. Sensory and assistive devices have long been developed to support patients in rehabilitation process [1]. One of the areas in which this technology can have a great impact is rehabilitation therapy. VR has many features that give it a unique rehabilitation potential, both as an intervention and as an assessment. With the help of VR, people can practice skills that could carry too much risk in the real world [2]. In a simulator, they can safely engage in a range of activities that can be beneficial to their health relatively free from the limitations [3]. Different studies suggest that VR therapy had a positive impact on the therapeutic modality for people, as it indicates benefits in activity and participation, brain reorganization, motor skills or visual-spatial outcomes, and personal factors [4].

Virtual systems have great potential as a technology and can provide a safe learning experience, especially for the rehabilitation of people with different limitations [5]. They allow various types of rehabilitation, as well as experimental and active learning that motivates and encourages patients. It also provides objective monitoring of behavior in a safe environment, while maintaining treatment control by monitoring stimuli and measuring time parameters [6]. It has significant potential to improve rehabilitation of users. Applications are being developed that can improve the effects of rehabilitation, life skills, quality of life, mobility, and cognitive abilities, and also act as a motivator on participants [7].

It can be seen from the literature that VR therapy is useful as a treatment for the rehabilitation of patients, and also can be a valuable addition to traditional programs [8]. In terms of outcomes, the findings of various studies show that VR rehabilitation improved skills, physical conditioning, and knowledge of individuals [9]. VR has credibility as a useful tool to teach persons independent living skills in a safe environment, with important repercussions on real life [10]. In rehabilitation therapy, a personal approach, where the therapeutic regime is adapted to specific patients can increase the chance of recovery. VR offers significant support for customizing patient experience. One of the potentials is to maintain cognitive functions by providing recovery training in patients with brain damage [11].

Even though VR technology has progressed over the past 20 years, users have reported feeling negative symptoms, similar to motion sickness, after exposure to virtual visual motion. The negative symptoms that VR users experience using the technology are known as cybersickness, and may sometimes lead to discontinuation of VR use [12].

Harington et al. have developed a medical training simulator and during testing, nausea was observed and noted during exposure to the VR due to uncoordinated visual and vestibular sensory stimulation [13]. Aldaba et al., to reduce simulator sickness during navigation through the VR, replaced the steering wheel with a modified wheelchair and concluded that wheelchairs significantly reduced the simulator sickness in users, but the need for a large space to maneuver and move through the virtual environment is a flaw of this experiment [14]. Fernandes and Feiner investigated the effects of dynamic, but subtle, changes in the user’s field of vision while in VR, the results showed that field of view restrictors help users use it longer and feel more comfortable [15]. In his master’s thesis, Tiiro tried to answer the question of whether higher visual realism causes more symptoms of cybersickness than low realism, and the results showed that a higher level of realism causes a higher degree of cybersickness than a lower level [16]. Kemeny et al. proposed a new navigation technique to reduce the incidence of cybersickness symptoms in users and found the appearance of stronger symptoms at faster rotations of the user’s visual field, and weaker at slower rotations [17].

The goal of this paper is to examine the influence of user navigation and moving elements and animations presented as dominant causes for the occurrence of cybersickness. As the culprits are identified, the focus will be on the ways the user navigates through VR and on the number of moving elements and animations. We will present a study that was conducted on a group of participants in a specifically designed VR environment. By changing and configuring different factors in the environment, the influence on the development of symptoms of cybersickness will become measurable. Identification of these factors can provide a valid input in the future design of virtual environments to reduce the occurrence of cybersickness.

### 1.1. Virtual Reality and History of Cybersickness

The term cybersickness was coined by McCauley and Sharkey in 1992 to describe the motion-sickness-like symptoms associated with VR characterized by “applications involving distant objects, that include, terrain, self-motion (travel) through the environment and the illusion of self-motion (vection) [18]. Cybersickness is a psychophysical response to the exposure of perceptual illusions in a VR. Unwanted symptoms can be divided into three categories: visual symptoms, disorientation, and nausea. Other symptoms that may occur during or after exposure to the VR may include: general discomfort, abdominal discomfort, belching, and sometimes vomiting, drowsiness, dizziness, headache, difficulty concentrating, blurred vision, and tingling in the eyes [19]. Symptoms may occur during exposure to a VR but may continue for some time after the end of the exposure.

Cybersickness is not a classical disease but is a psychological response of the human organism to an unusual stimulus. It is a rather common occurrence for people exposed to VR. Although different symptoms and their frequency have been documented, research shows that oculomotor problems usually outweigh the human response to VR. The capabilities and limitations of users can significantly affect the effectiveness and usability of the VR. Due to the impact of the VR on the experience of participants, the problems that arise when using it need to be considered from the early stages of its design. For example, the designers of a VR system should bear in mind that the human body is limited by sensory, perceptual, and motor limitations. These factors influence the development of certain symptoms and can be modified to reduce the negative effects on the user.

To specifically differentiate cybersickness as an occurring condition, we must clearly define what constitutes VR. Electronic environment simulations experienced via a head-mounted display that enables the end-user interaction in realistic three-dimensional situations is considered VR [20]. These simulations enable users to interact with an artificial 3D visual or another sensory environment by the use of computer simulation and modeling [21]. A VR system forms a stereo pair delivering a left- and right-eye image with the use of an active stereo system in which there is no leakage of the left-eye image to the right eye, and vice versa. The images are updated in real-time and generated in the graphics pipeline of a computer system [22].

The problems that are related to the VR system nowadays are similar to the problems that appeared at the very beginning of the mass use of passive transport, so it is necessary to analyze this phenomenon as well. The development and increased use of ships once pointed to the existence of seasickness problems. The oldest record of seasickness is mentioned in Hippocrates’ Aphorisms [23]. from 2400 years ago. Similar symptoms were later observed on land. Napoleon’s scouts in Egypt, who used camels as a means of transportation, contracted motion sickness [24].

The development of computers has contributed to the possibility of creating simulations in which the user is in an improvised cabin with screens instead of windows, so these environments cause conflicts of the human sensorimotor system and cause certain side effects. The use of such simulators has shown problems with symptoms very similar to motion sickness. This type of negative side effect is called simulator sickness [25]. Taking into account this discovery, it is clear that motion sickness is not only related to movement but also occurs when creating the illusion of passive movement in a person. Due to this conclusion, it is not surprising to learn that later VR systems create similar problems and symptoms in users. This type of sickness, although it has similar symptoms as the others listed, differs due to its appearance during a stay in cyberspace and is called cybersickness [26].

### 1.2. Characteristics of Virtual Environments

#### 1.2.1. Immersion

The more the system provides users with a display (in all sensory modalities) with the credibility of tracking transferred from the real world, the more it is “immersed” [27]. Immersion in VR is the perception of physical presence in the nonphysical world [28]. The level of immersion in the VR depends on the system rendering software and display technology (including all types of touch screens). Given that immersion is objective and measurable, one system may have a higher level of immersion than another [29].

#### 1.2.2. Presence

Presence is an abbreviated term derived from the word “telepresence”. It is a psychological state or subjective perception in which part or all of an individual’s current experience is generated and/or filtered by technology, but where part or all of an individual’s perception fails to accurately recognize the role of technology in that experience [30]. The sense of presence is defined as the subjective experience of being in one place or environment, even when a person is physically in another [31]. The presence for that reason requires that the participants identify with the virtual bodies, that they consider their movements as their own, and that the virtual body is the real body of that person in the virtual world [32].

#### 1.2.3. Vection

When someone is in a stationary position, under certain conditions he can still get the impression of self-movement. This deceptive impression of self-movement is called vection [33]. It is generally accepted that large field of view screens covering peripheral vision is most effective in creating a vection [34]. Vection is important for VR because it enhances realism, presence, or the user experience [35]. Vection is a feeling of self-movement caused by visual stimulation, so more vection creates more sensory conflict. Conditions that increase vection also increase cybersickness [36].

### 1.3. Types of Negative Symptoms and Effects

Motion sickness, simulator sickness, and cybersickness have similar symptoms but are caused by exposure to slightly different situations. Motion sickness is an uncomfortable feeling, often accompanied by nausea, dizziness, and vomiting that can occur when people travel in moving vehicles [37]. Although there are definite relationships between the symptoms that occur in motion sickness, simulator sickness, and cybersickness, different clusters of symptoms can be found that distinguish the three conditions, as identified in Table 1 [38].

The authors Kennedy et al. [25]. identified 27 symptoms experienced by users in a series of factor analyses. Eliminating symptoms that had a low incidence rate, the authors developed and validated a new Simulator Sickness Questionnaire (SSQ) containing 16 symptoms (Table 2).

### 1.4. Cybersickness as Obstacle in Using VR for Rehabilitation of Patients

The VR community, during the past years, has based its development on a synthesis of earlier work in interactive 3D graphics, visual simulation, and user interfaces [39]. How users interact with different systems has changed over time [40]. An increasing number of people are accessing multimedia content daily using a variety of devices, such as computers, televisions, and smartphones, and each of them can play virtual content [41]. Therefore, it is important to have a degree of knowledge about the current use of VR technologies in the field and to explore the possible impacts such technologies have on users [42]. Although this technology is experiencing rapid development and growing interest, at the same time the observed negative symptoms and effects that occur in users when interacting with the VR present a cause for concern. These problems can negatively affect the use of virtual systems in the rehabilitation, as systems designed to provide assistance and recovery can cause additional negative symptoms for users. Taking these facts into account, cybersickness and its impact on users is one of the primary problems that need to be solved for the sake of safely using virtual systems in rehabilitation processes. The negative impact that the VR has on the experience of participants and problems that may arise when using the VR system itself, should be considered from the earliest stages of its design because user capabilities and limitations can significantly affect the effectiveness and usability of the application.

## 2. The Process of Creating and Testing the Impact of a Virtual Environment on a User

### 2.1. Setting of Test Parameters

In this paper, we tested the influence of interaction between humans and computers through navigation in VR, the amount of animation and moving elements in the environment, and their overall impact on the occurrence of cybersickness. The aim was to cross-reference and test two ways of movement with two types of animations and moving elements in the environment to provide a clear insight into their impact while interacting in a VR.

Users stood upright and had full 360 degrees of rotation freedom by physically turning around their axis. This movement was tracked by five cameras located in an Oculus head-mounted display. Oculus controllers were used for navigating through the VR. Two common methods of navigation in VR were tested: natural locomotion and teleporting [43].

**Figure 1:**
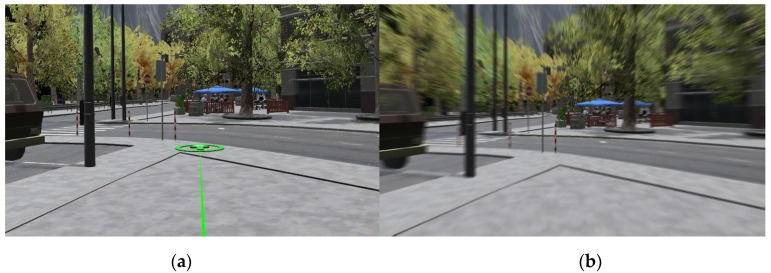
Environment navigation via teleport (**a**) and navigation via natural locomotion movement (**b**).

**Figure 2:**
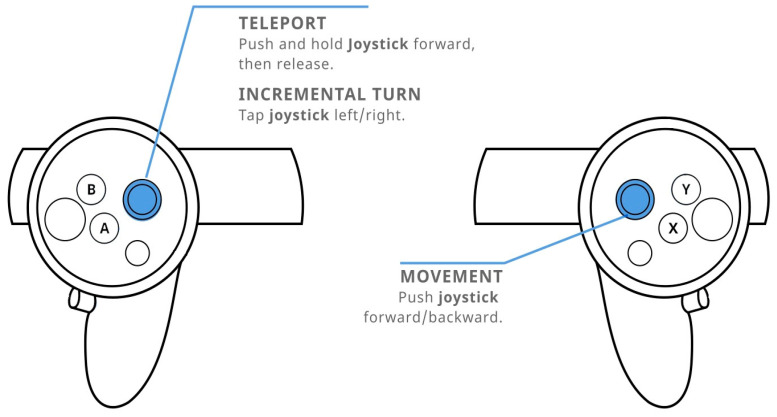
Oculus thumbstick navigation commands via teleport (**left**) and navigation via natural locomotion movement (**right**) [44].

Considering navigation methods (Figure 1 and Figure 2), these are two types commonly used to interact with a complex 3D environment and don’t require expensive technologies and a large space for installing required equipment e.g., VR treadmill. Teleport navigation is a way of navigation in which the user points the controller towards the place where he wants to move and at the press of a button, he simply appears at that place. In addition to real-life head rotation, users can rotate left or right by pushing the thumbstick in that direction. Natural locomotion movement is a way of navigation control where the user moves in virtual space by using the thumbstick on the Oculus touch controllers but rotates by turning their head or whole body in physical space around them, simulating the real-life movement. This allows them to move in any direction and to adjust the speed of movement by the level of pressure they apply to the thumbstick.

**Figure 3:**
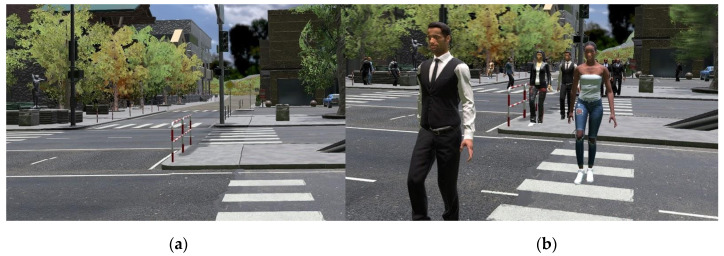
Environment without animation and moving elements (**a**), environment with animations and moving elements (**b**).

Two types of environments were also tested (Figure 3), one of which was static, and had no animations and moving elements, while the other was rich in animations and moving elements. In the first, static, environment, there was no movement of vegetation in the environment which was caused by the intensification of the influence of the wind, as well as passersby moving through the environment. The second environment, unlike the first, had a strong movement of vegetation, due to the increased influence of the wind and a large number of passersby moving through the environment. The trees were located on the sidewalks all over the city as to be most of the time in the user’s field of view. There were eight people per node, and nodes were set on each street junction, each in a random direction and offset movement route.

**Figure 4:**
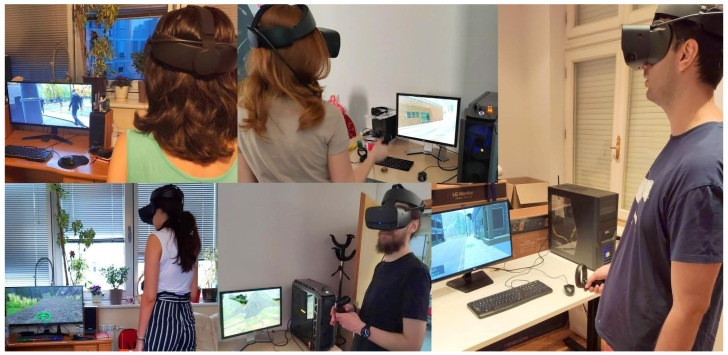
Testing the virtual simulation.

In all four tests, (Figure 4) users had complete freedom of movement through the created VR environment via controllers and stated ways of movement, while the physical movement was limited to one meter in circumference around the user. This enabled them to fully feel the impact that changes in the environment and the navigation have on their psychophysical condition and the appearance of cybersickness.

### 2.2. Testing and Collecting Results

Fifty respondents participated in the research. Data were collected in a period of 44 days, from 24 June to 6 August 2020. Twenty-one women and twenty-nine men participated in the survey, representing 58% of men and 42% of women. Ages ranged from 24 to 45 years. In this way, the factor of the influence of age was excluded, considering that children and persons older than 55 are less susceptible to the appearance of cybersickness [45]. Twenty-four years was the minimum for both genders, while the maximum for female respondents was 43 years, which is two years less than for men, where the maximum number was 45 years. When it comes to experience and previous use of the VR system, 33 respondents had never used such a system before this testing, while 17 respondents had encountered it before, but as a single-use and not continuous long-term usage. In percentages, that constitutes 34% of respondents that have used some virtual system before, and 66% of respondents that have not.

A representative sample of respondents was tested on each of the four environments for four minutes, and, after each test, they filled out a questionnaire on negative symptoms and effects on the human body caused by the different environmental characteristics and user interactions. Users tested the virtual simulation while standing and had 360-degree rotation freedom. They moved through the environment via the thumbstick, the right thumbstick set for teleport navigation, and the left for natural locomotion movement. Depending on the need of the test, users were allowed to use one of the two explained movement modalities throughout the environment.

Users first tested the environment without animations and moving elements in the environment, and moved via teleport. The second environment they tested was with strong animations and many moving elements with the same movement modality. The third test was in an environment without animation, but with natural locomotion movement. The last test was with natural locomotion movement and with a lot of animations and moving elements in the environment.

This test schedule was chosen because the first environment had the fewest elements that could cause problems in use, so it was appropriate for users to get an impression of the environment and how to evaluate it. Further on, each subsequent test environment introduced more elements. One week was given between each test for rest. This approach was chosen because the repetitive experience itself could lower the sensibility and make the users adapt to the usage of VR resulting in the decrease of symptoms [24].

The Simulator Sickness Questionnaire (SSQ) developed by Kennedy et al. in 1993 was used to collect the data. The questionnaire contained 16 questions, all listed in Table 3, and related to various symptoms that may have occurred during an interaction within a VR. The mentioned 16 questions were divided into three groups of symptoms: nausea, oculomotor symptoms, and disorientation. A five-point Likert [46] scale was used to assess the severity of symptoms.

### 2.3. Creating Virtual Environment

In pursuit of finding key interaction elements that can cause cybersickness, the adaptive VR environment had to be defined for this study. In that process, the decision was to use several development applications to create the VR environment. The basis of the software solution used for the study would be a VR environment created in the Unity 3D program. Modeling of the environment and elements was done in the Blender program, and the GIMP program was used to create the textures of the objects. The user interaction and the logic of the environment were managed by scripts written in the C# programming language.

For this research, in addition to general scientific methods, the following procedures were applied:

- the modeling of the VR environment;
- model testing and filling in questionnaires by users;
- analytical-deductive and statistical methods.

**Figure 5:**
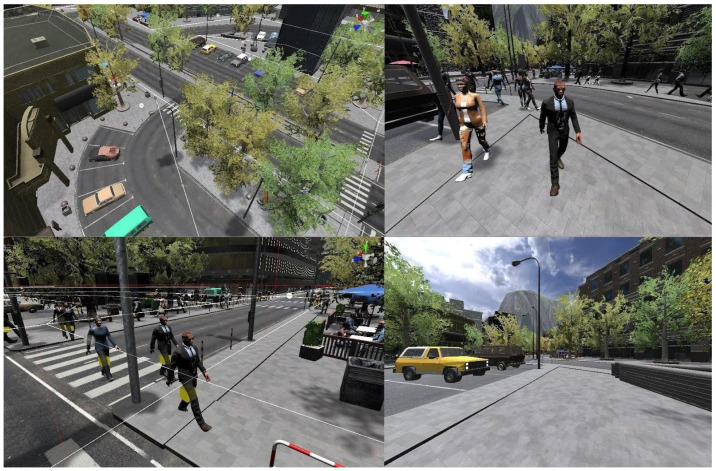
Virtual environment for testing.

Modeling, texturing, and animation were used to create a VR environment for testing purposes (Figure 5). The VR environment was modeled in four ways with different characteristics aiming to examine all parameters relevant to the subject of research. They were also modeled to eliminate the factors that could have an impact on the onset of symptoms not relevant to the study. The analytical-deductive method was used to analyze the data obtained through the questionnaire.

## 3. Evaluation of the Obtained Results

In Table 3, we can see that the first test, without animations and with teleport as a way of navigation, caused the least problems for all users. The second test, which also used teleport as a way of navigation, but had moving elements and animations in the environment, caused some more problems. The third test shows a significant jump in the overall results.

Taking into account that this was a test with natural locomotor movement, and without animations and moving elements, we can conclude that this way of navigation increases the problem. The last test, natural locomotor movement with animations and moving elements in the environment, caused the most problems for users when interacting in VR.

**Figure 6:**
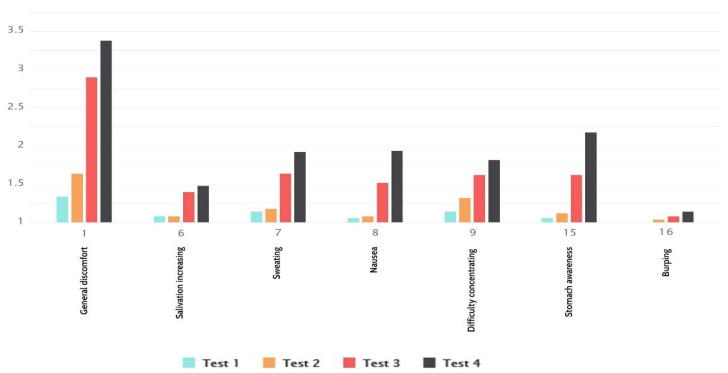
Nausea results.

If the questions related only to nausea were singled out (Figure 6), we could see that the results are in line with the total results, and lower than the total values. This means that fewer respondents felt a stronger presence of nauseating effects. General discomfort was a symptom with the highest value, and burping had the lowest reported value, while other symptoms had similar values.

It stands out that the natural locomotion type of navigation had much higher values than teleport. We can also see that there was a difference in values regarding environment animation, where no animation environment had slightly lower values.

**Figure 7:**
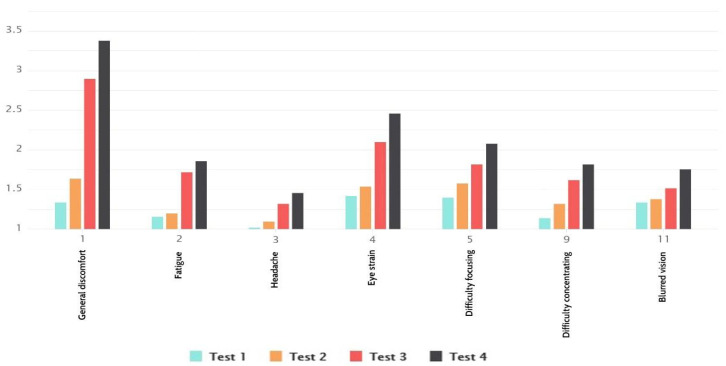
Oculomotor symptoms results.

From the of oculomotor symptoms (Figure 7), we concluded that the first two tests had higher average scores than total results, but interestingly, at the same time, the other two tests had lower average values. We noticed that general discomfort had the highest reported values. The question regarding vision follow-up and headache had the lowest reported values. We perceived that there was a larger difference in symptom occurrence with types of navigation than environment animation and moving elements. Following, it was noted that difficulty focusing and blurred vision had the smallest difference between types of navigation and environment moving elements.

**Figure 8:**
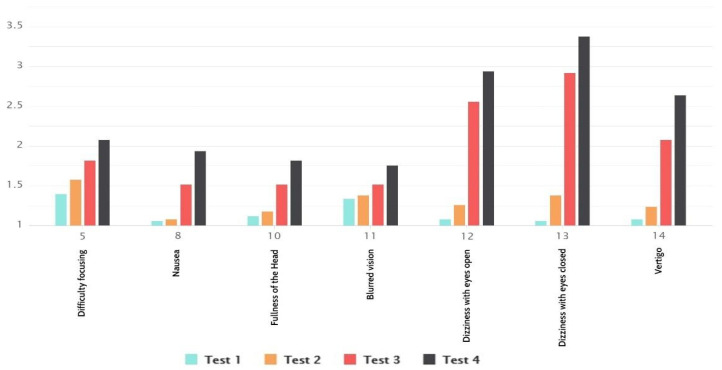
Disorientation results.

The disorientation table (Figure 8), shows higher average values than the average values in the overall results, except for the average values from the second test. We could see that dizziness and vertigo had higher values, whereas there were larger differences in the type of navigation levels than other symptoms. It was noticeable that difficulty in focusing and blurred vision singled out as the ones sensitive to the types of navigation and moving elements and animations.

**Figure 9:**
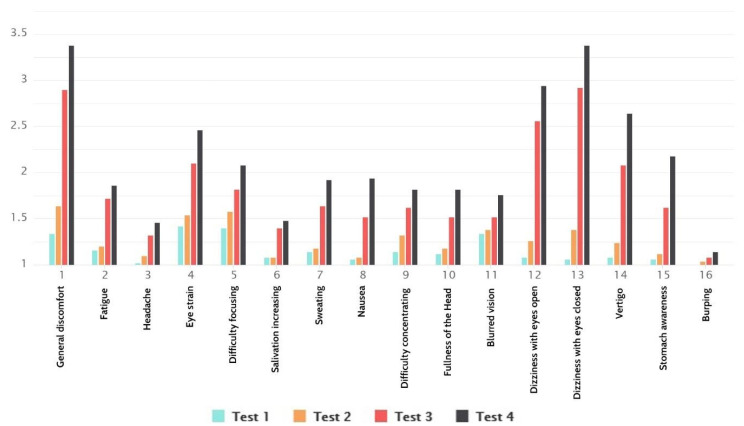
Total Simulator Sickness Questionnaire (SSQ) results.

From the table of total average results (Figure 9), we understood that the least problems when using the VR system were related to nausea, which had the least recorded average values in all tests. The disorientation caused the most problems in tests with natural locomotor movement, especially in combination with animations and moving elements. Most problems with the oculomotor system were manifested in tests with teleportation as a way of navigation, and especially with the added animation and moving elements. The general discomfort, dizziness, and vertigo portrayed the highest values, while burping had the lowest. There was again a notable difference in values between navigation types.

**Figure 10:**
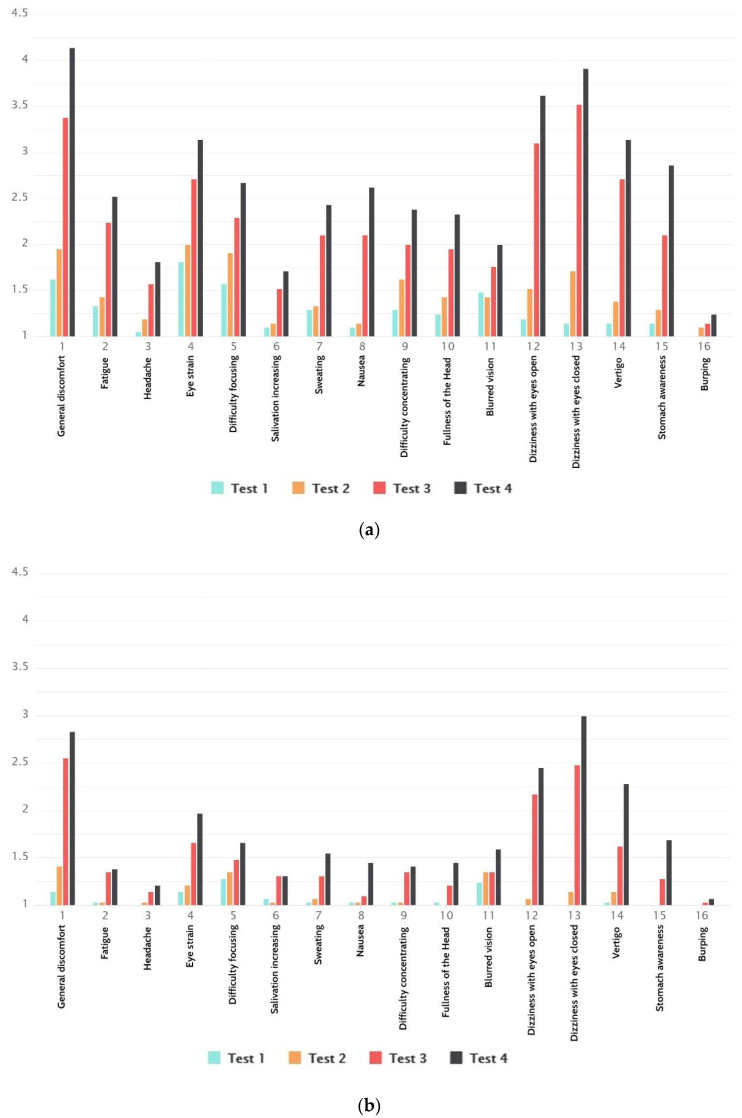
(**a**) Total SSQ results for women. (**b**) Total SSQ results for men.

From Figure 10a,b, we can see that women and men did not react the same to the appearance of cybersickness. Women were more susceptible and had pronounced symptoms. Even though the results show cybersickness as gender-sensitive, the results were in line with total results for both men and women.

As stated previously, there were four independent tests conducted by each participant. Their subjective feeling was recorded after each test using standardized questionnaires applied after every test. In order to be able to conclude from the retrieved results, we initially had to interrogate the validity of the retrieved results. For this purpose, we have conducted a one-way ANOVA for each SSQ subscale and the total SSQ score on all four tests (Table 4). ANOVA results for total SSQ score show that M = 1.6, SD = 0.6 and f-ratio value 17.8131. The *p*-value is <0.00001. The *p*-value corresponding to the F-statistic of one-way ANOVA is lower than 0.05, suggesting that one or more treatments are significantly different.

To proceed, we decided to compare results between all four of our cybersickness tests. We applied Tukey’s [47] honestly significant difference (HSD) test to each of the six combination pairs to pinpoint the one that exhibits significant statistical differences. As results indicated (Table 5), there were no significant differences between tests 1 and 2, as well as tests 3 and 4, and these test pairs had the same navigation method.

On the other hand, there were significant differences between all other tests. Results for other test pairings, with different navigation methods, showed significant differences and proved that navigation methods had more influence in cybersickness occurrence than animation and number of moving objects for the total score. From this part of the research, we can conclude that navigation has the most influence on symptom occurrence, and locomotion navigation mostly induces more side effects than teleport.

After determining the significance of results between tests, we set out to interrogate the link between our tests and the influence they exerted on groups of symptoms. In this study, three groups of symptoms were classified: nausea, oculomotor symptoms, and disorientation.

First in line was nausea, (Table 6) where ANOVA was initially conducted to determine significance among tests.

ANOVA results for nausea symptoms showed that M = 1.5, SD = 0.57, the f-ratio value 5.2448 and the *p*-value of 0.0063. The result is significant at *p* < 0.05.

Within the question group that was related to symptoms of nausea (Table 7), we had completed across test comparison using Tukey HSD. Results indicated that there were significant differences in Tukey’s test between tests 1 and 4, as well as 2 and 4. Based on that we concluded that nausea was mostly induced with a combination of natural locomotion navigation with animations and moving elements. Alternative navigation via teleport could not be considered as a strong enough inhibitor of this symptom. All other test combinations showed insignificant differences in cybersickness occurrence.

From ANOVA results (Table 8) for oculomotor symptoms, the results obtained were M = 1.66, SD = 0.54, and the f-ratio value 6.0356. The *p*-value came to 0.0033. The result is significant at *p* < 0.05.

Within the question group that was related to oculomotor symptoms (Table 9), Tukey’s test showed similar results as nausea. Results indicated that only test 4, with natural locomotion as navigation method and with animations and moving elements, had significant differences from tests 1 and 2 that had teleport as a means of navigation. We concluded that oculomotor symptoms were mostly induced with a combination of natural locomotion navigation with animations and moving elements. All other test combinations showed insignificant differences in cybersickness occurrence.

Disorientation ANOVA results (Table 10) showed that M = 1.7, SD = 0.65 and the f-ratio value of 12.0602. The *p*-value is 0.0001. The result is significant at *p* < 0.05.

Within the question group that was related to disorientation symptoms (Table 11), test results in Tukey’s test showed similar results as the total score. It proved that tests with different navigation methods had significant differences. Disorientation is mostly induced with natural locomotion as a type of navigation, so animations with moving elements cannot be considered as a strong enough inhibitor of this symptom. All other test combinations showed insignificant differences in cybersickness occurrence.

## 4. Discussion

The work on this article starts from the very essence of the problem that can occur when using VR systems in rehabilitation. The results indicate the existence of the problem and its impact on the acceptance of virtual systems as a way of rehabilitation for users. As the contemporary literature designates the benefits of VR in the rehabilitation process, this paper contributes by outlining the existence of problems and offer solutions to ease the use of VR systems.

This research was aimed to determine navigation methods and animation and number of moving objects as factors of human–computer interaction in VR that have a strong influence on the occurrence of cybersickness. The focus was on the following aspects: the way users move through VR as well as the amount of static or moving elements in VR.

For this purpose, we designed and conducted a study that included 50 participants with different professional backgrounds. None of them owned VR systems or used them extensively. None of the testers had advanced knowledge in using and navigating through VR or had been instructed how to use it.

The results revealed an indication of a link between the way of navigating through the VR with the occurrence of cybersickness. The test showed that natural locomotion navigation that depicts real movement produced more symptoms of cybersickness than navigating via teleport. At the same time, the number of moving elements and animation in the environment increased the level of severity of cybersickness in people. A static environment without moving elements caused fewer symptoms than an environment with moving elements and animations.

General discomfort, eye strain, and focusing difficulty had the highest levels of symptoms, followed by blurred vision, in the first two tests where users navigate through VR via teleport. Two-second tests, with natural locomotion type of navigation, general discomfort, and dizziness, both with eyes open and closed, had the highest levels of cybersickness symptoms.

Gender sensitivity came out as interesting test results, as it show that women are more susceptible to cybersickness than men. Some researchers suggest that this is because women naturally have a wider field of vision than men, and a wide field of view increases the likelihood of flickering perception [33]. At least three possible explanations are given: hormonal differences, differences in visual field width, and biased self-report data. The hormonal hypothesis is the same as that given in the motion sickness literature—that women are more susceptible to simulator sickness during part of the menstrual cycle [48].

## 5. Conclusions

In conclusion, the navigation mode and animation and moving elements influence the use of VR systems. Natural locomotor navigation type causes more cybersickness symptoms in these tests than teleport, as confirmed by results in all symptom groups and total score. Environment animations and moving elements do not show significant differences in the level of cybersickness symptoms provoked when compared with static and nonmoving environments. However, these will probably increase the side effects and thus negatively influence the user’s experience.

This study has shown that there is a possibility to reduce the occurrence of problems related to cybersickness if the results of this research are taken into consideration and applied from an early stage of designing a VR.

When designing a VR simulation for rehabilitation, the recommendation is to use teleport as a way of navigation or to combine teleport and natural locomotion if there is a possibility, because there is a probability to lower cybersickness symptoms with lesser use of natural locomotion as a way of navigation. It is recommended to find a balance between the number of animated and moving elements, as well as the needs and realism of the environment itself. As results show moving and animated environments as triggers of the cybersickness symptoms, the solution would be to reduce these negative effects by eliminating or lowering the number of animated and moving elements.

Appreciation of the complex interactions among and between contributing factors can lead to the development of a predictive model [49]. People could benefit greatly from multisensory environments when it comes to rehabilitation and their recovery [50]. Cybersickness is a complex problem that can reduce the effectiveness of the virtual application itself and cause certain health problems during exposure to VR [51]. Depending on the interaction of a particular participant in the VR, the system must be designed to improve the performance of that user, stimulate his participation in rehabilitation and avoid giving up or creating aversion [52]. Factors affecting the efficiency of human work in VR represent a complex problem that can reduce effectiveness and cause certain health problems during exposure to VR [53]. The ability to more accurately predict this phenomenon contributes to more efficient and effective work in creating VR for the rehabilitation.

Rehabilitation users of virtual simulations can greatly benefit from this research results, by learning about the possibility of certain symptoms occurring in the virtual space. Being prepared and able to recognize the symptoms before they immerse in the virtual world could prevent the aversion in the future use of VR rehabilitation applications. Understanding the causes and factors that influence the occurrence of these negative symptoms and effects strongly contributes to building a procedure in VR system development, which would improve human–computer interaction when it comes to using a virtual rehabilitation system.

## Figures and Tables

**Table 1:** Symptoms [38].

Nausea	Oculomotor Symptoms	Disorientation
Stomach discomfort Increased salivation Burping	Tingling in the eyes Loss of concentration Blurred vision A headache	Dizziness Loss of balance

**Table 2:** Kennedy classification of symptoms [25].

General Discomfort	Difficulty Concentrating
Fatigue	Fullness of the Head
Headache	Blurred vision
Eye strain	Dizziness with eyes open
Difficulty focusing	Dizziness with eyes closed
Salivation increasing	Vertigo
Sweating	Stomach awareness
Nausea	Burping

**Table 3:** Overall results.

Symptom	Test 1 Mean (SD)	Test 2 Mean (SD)	Test 3 Mean (SD)	Test 4 Mean (SD)
1. General discomfort	1.34 (0.52)	1.64 (0.60)	2.90 (0.91)	3.38 (1.18)
2. Fatigue	1.16 (0.42)	1.20 (0.45)	1.72 (0.83)	1.86 (1.05)
3. Headache	1.02 (0.14)	1.10 (0.36)	1.32 (0.84)	1.46 (0.86)
4. Eye strain	1.42 (0.57)	1.54 (0.68)	2.10 (0.93)	2.46 (1.13)
5. Difficulty focusing	1.40 (0.53)	1.58 (0.64)	1.82 (0.98)	2.08 (0.93)
6. Salivation increasing	1.08 (0.27)	1.08 (0.34)	1.40 (0.76)	1.48 (0.93)
7. Sweating	1.14 (0.49)	1.18 (0.44)	1.64 (0.96)	1.92 (1.31)
8. Nausea	1.06 (0.31)	1.08 (0.34)	1.52 (0.84)	1.94 (1.11)
9. Difficulty concentrating	1.14 (0.40)	1.32 (0.55)	1.62 (0.85)	1.82 (0.92)
10. Fullness of the head	1.12 (0.38)	1.18 (0.48)	1.52 (0.76)	1.82(1.04)
11. Blurred vision	1.34 (0.74)	1.38 (0.75)	1.52 (0.86)	1.76 (1.08)
12. Dizziness with eyes open	1.08 (0.34)	1.26 (0.56)	2.56 (0.95)	2.94 (1.25)
13. Dizziness with eyes closed	1.06 (0.31)	1.38 (0.70)	2.92 (0.90)	3.38 (1.07)
14. Vertigo	1.08 (0.34)	1.24 (0.51)	2.08 (1.00)	2.64 (1.21)
15. Stomach awareness	1.06 (0.31)	1.12 (0.38)	1.62 (0.83)	2.18 (1.08)
16. Burping	1 (0)	1.04 (0.28)	1.08 (0.44)	1.14 (0.49)
The cumulative result	1.13 (0.12)	1.31 (0.25)	1.90 (0.60)	2.25 (0.78)

**Table 4:** Total SSQ score ANOVA summary.

Source	Sum of Squares SS	Degrees of Freedom ν	Mean Square MS	F Statistic	*p*-Value
test	10.4545	3	3.4848	17.8131	2.1989 × 10^−8^
error	11.7379	60	0.1956		
total	22.1924	63			

**Table 5:** Total SSQ score Tukey HSD results.

Test	Tukey HSD Q Statistic	Tukey HSD *p*-Value	Tukey HSD Inference
1 vs. 2	1.0287	0.8766316	insignificant
1 vs. 3	6.1270	0.0010053	*p* < 0.01
1 vs. 4	8.9079	0.0010053	*p* < 0.01
2 vs. 3	5.0983	0.0034737	*p* < 0.01
2 vs. 4	7.8792	0.0010053	*p* < 0.01
3 vs. 4	2.7809	0.2122966	insignificant

**Table 6:** Nausea SSQ score ANOVA summary.

Source	Sum of Squares SS	Degrees of Freedom ν	Mean Square MS	F Statistic	*p*-Value
test	3.4672	3	1.1557	5.2448	0.0063
error	5.2886	24	0.2204		
total	8.7558	27			

**Table 7:** Nausea SSQ score Tukey HSD results.

Test	Tukey HSD Q Statistic	Tukey HSD *p*-Value	Tukey HSD Inference
1 vs 2	0.5153	0.8999947	insignificant
1 vs 3	3.1885	0.1373060	insignificant
1 vs 4	4.8632	0.0107602	*p* < 0.05
2 vs 3	2.6732	0.2587072	insignificant
2 vs 4	4.3479	0.0249842	*p* < 0.05
3 vs 4	1.6748	0.6288279	insignificant

**Table 8:** Oculomotor SSQ score ANOVA summary.

Source	Sum of Squares SS	Degrees of Freedom ν	Mean Square MS	F Statistic	*p*-Value
test	3.3489	3	1.1163	6.0356	0.0033
error	4.4389	24	0.1850		
total	7.7878	27			

**Table 9:** Oculomotor SSQ score Tukey HSD results.

Test	Tukey HSD Q Statistic	Tukey HSD *p*-Value	Tukey HSD Inference
1 vs 2	0.8261	0.8999947	insignificant
1 vs 3	3.6736	0.0701518	insignificant
1 vs 4	5.2732	0.0053753	*p* < 0.01
2 vs 3	2.8475	0.2111234	insignificant
2 vs 4	4.4470	0.0213151	*p* < 0.05
3 vs 4	1.5995	0.6580020	insignificant

**Table 10:** Disorientation SSQ score ANOVA summary.

Source	Sum of Squares SS	Degrees of Freedom ν	Mean Square MS	F Statistic	*p*-Value
test	6.8357	3	2.2786	12.0602	5.1824 × 10^−5^
error	4.5344	24	0.1889		
total	11.3701	27			

**Table 11:** Disorientation SSQ score Tukey HSD results.

Test	Tukey HSD Q Statistic	Tukey HSD *p*-Value	Tukey HSD Inference
1 vs. 2	0.8348	0.8999947	insignificant
1 vs. 3	5.0434	0.0079470	*p* < 0.01
1 vs. 4	7.3216	0.0010053	*p* < 0.01
2 vs. 3	4.2086	0.0311513	*p* < 0.05
2 vs. 4	6.4869	0.0010053	*p* < 0.01
3 vs. 4	2.2782	0.3927065	insignificant

## Data Availability

The data presented in this study are available on request from the corresponding author. The data are not publicly available due to privacy reasons.
